# International harm reduction indicators are still not reached: results from a repeated cross-sectional study on drug paraphernalia distribution in Germany, 2021

**DOI:** 10.1186/s12954-023-00870-2

**Published:** 2023-09-19

**Authors:** Franziska Hommes, Amrei Krings, Achim Dörre, Esther Neumeier, Dirk Schäffer, Ruth Zimmermann

**Affiliations:** 1 Department of Infectious Disease Epidemiology, Robert Koch Institute, Berlin, Germany; 2 Postgraduate Training for Applied Epidemiology (PAE), Robert Koch Institute, Berlin, Germany; 3 ECDC Fellowship Programme, Field Epidemiology Path (EPIET), European Centre for Disease Prevention and Control (ECDC), Stockholm, Sweden; 4 IFT Institut Für Therapieforschung, Centre for Mental Health and Addiction Research, Munich, Germany; 5 German AIDS Service Organization, Berlin, Germany

**Keywords:** Intravenous drug users, Blood-borne infections, Harm reduction, Surveillance

## Abstract

**Background:**

To prevent the transmission of blood-borne infections and reach the elimination of viral hepatitis by 2030, the World Health Organization (WHO) has set the goal to distribute 300 sterile needles and syringes each year per person who injects drugs (PWID). We aimed to assess drug paraphernalia distribution in Germany in 2021, including the WHO indicator, and to analyse changes to the distribution measured in 2018.

**Methods:**

We conducted a repeated cross-sectional study of low-threshold drug services in Germany. We assessed type and quantity of distributed drug paraphernalia and the number of supplied PWID in 2021 using an online and paper-based questionnaire. We conducted a descriptive statistical analysis of data from 2021, assessed fulfillment of the WHO indicator and changes in services that participated 2021 and in the previous study 2018.

**Results:**

Five hundred and eighty-nine of 1760 distributed questionnaires were returned in 2021. 204 drug services from 15 out of 16 federal states confirmed drug paraphernalia distribution, covering 20% of Germany’s rural and 51% of urban counties. 108 services had also participated in 2018. The most frequently distributed paraphernalia for injecting drug use in 2021 were syringes (97% of services), needles (96%) and vitamin C (90%). Pre-cut aluminium foil (79% of services) and pipes (28%) for inhaling, and sniff tubes (43%) for nasal use were distributed less frequently. We found a median reduction in distributed syringes by 18% and by 12% for needles compared to 2018. Of 15 states, two reached the 2030 WHO-target for needles and one for syringes.

**Conclusions:**

The current national estimates and changes from 2018 to 2021 for drug paraphernalia distribution seem far from meeting the WHO target. Reasons could include a change in drug consumption behaviour towards less injecting use and more inhaling, and effects of the COVID-19 pandemic (supply difficulties, social distancing, lockdown, reduced opening hours of services). We observed pronounced regional differences in drug paraphernalia distribution. To close existing gaps, Germany should expand its drug paraphernalia distribution programmes and other harm reduction services, such as drug consumption rooms. Further investigation of determinants for adequate distribution is essential to reduce blood-borne infections in this key population.

**Supplementary Information:**

The online version contains supplementary material available at 10.1186/s12954-023-00870-2.

## Background

The use of sterile needles, syringes and other drug paraphernalia is part of a comprehensive package to prevent bloodborne infections in people who inject drugs (PWID) [1, 2]. To reduce the risk of HIV and viral hepatitis infections and transmissions through the use of non-sterile injection equipment, the World Health Organization (WHO) set the number of annually distributed needles and syringes per PWID as an indicator in its Global health sector strategies 2022–2030 on HIV, viral hepatitis and sexually transmitted infections, with a target of 300 sterile needles and syringes (n/s) per PWID by 2030 (previous target: 200 n/s per PWID until 2020) [1].

In Germany, interventions for harm reduction are part of the national drug strategy [3] and the need for an adequate supply of n/s for PWID has been addressed in Germany’s integrated strategy for HIV, Hepatitis B and C and other sexually transmitted infections [4].

Previous national and international studies have shown that the risk of using non-sterile drug paraphernalia is associated with inadequate access to services providing sterile equipment [5–7]. Results from systematic reviews on the effectiveness of harm reduction interventions, such as needle and syringe programmes, suggest that such interventions might be effective both for the reduction of HIV and HCV transmission [8, 9]. For low dead space (LDS) n/s, a type of needles and syringes that retain a reduced amount of fluid (e.g. blood) after injection, a substantial reduction in the risk of HIV transmission [10, 11] and cost-effectiveness [12] have been shown.

Besides n/s, access to other sterile drug paraphernalia for injecting drug consumption are also relevant to reduce the risk of bloodborne infections and potential infection transmission [13]. These drug paraphernalia other than n/s include e.g. water [14], filter [15, 16] and cookers (synonym: spoons) [16], which impose a risk of infection if not used in sterile form. As an alternative, aluminium foil is used for inhaling/smoking heroin and reduces the risk of overdosing and viral infections compared to injecting use [17]. Additional items such as nasal ointment, vein ointment and lubricant gel can reduce the risk of skin and mucosa injuries and thus the risk of infection transmission but can also be a source of infection transmission if being shared.

The German AIDS Service Organization (in German: *Deutsche Aidshilfe*) has published recommendations on the distribution of drug paraphernalia, which include low-threshold access to harm reduction services, the supply of a variety of different drug paraphernalia and their distribution free of charge according to the individual need of clients [18].

Access to sterile drug paraphernalia for PWID in Germany is provided by a variety of drug services, including low-threshold services, drug consumption rooms, housing projects, n/s vending machine, and drug counselling centres [19]. In Germany, the operation of the services are the responsibility of the federal states and municipalities. Currently, drug consumption rooms, for which the operation is regulated by law on federal state level (§ 10a BtMG), exist in half of Germany’s 16 federal states.

Systematic and continuous data collection on the number of sterile n/s distributed per PWID in Germany is scarce [20]. An initial survey regarding the number of distributed n/s and other drug paraphernalia was performed in 2018 including the responses from 155 drug services in Germany [21].

In this study, we aimed to assess the current status and changes between results for 2018 and 2021 on the distribution of drug paraphernalia in Germany, in order to monitor progress towards the WHO target and inform future harm reduction policies.

## Methods

We conducted a repeated cross-sectional study of drug services in Germany between March and August 2022. The current study is a follow-up of a first study assessing drug paraphernalia distribution in Germany in 2018. The methodology of the first study is described in detail by Zimmermann et al. [21]. We contacted more than 1760 drug services from a database compiled for the first study, which was based on an online repository of drug services in Germany [22] and additions by the study group. The database included organizations with more than one service attached and individual low-threshold services (including outreach), drug consumption rooms, other low-threshold services and drug counselling centres. For the follow-up study, we cross-checked the database for duplicates and invalid contact information.

The study questionnaire consisted of 13 closed questions covering the following topics: (1) geographical location of the drug service, (2) the types and quantities of drug paraphernalia distributed, (3) the number of supplied PWID in 2021, (4) the sites and modes of distribution (multiple answers possible), (5) the costs at which needles and syringes were distributed, and (6) the budget available for the distribution of drug paraphernalia in 2021. The services were asked to state if the indicated number of distributed drug paraphernalia and supplied PWID were based on exact numbers or on estimates to better assess the validity of this data. The questionnaire did not include a specific definition of PWID, e.g. regarding the frequency of drug consumption in 2021.

We defined low-threshold services as drug services that offer access to PWID without any prerequisites (i.e. administrative or financial barriers, being abstinent), often conducting outreach activities. Drug consumption rooms offer different modes of drug consumption in a controlled and hygienic environment, often combined with counselling and social services. In contrast, we considered drug counselling centres and housing projects (not including homeless shelters) as services requiring any kind of organizational effort by the PWID, such as registration, an appointment or referral.

We defined urban and rural counties according to the division of regional authorities in Germany (in German: *Stadtkreise/kreisfreie Städte* and *Landkreise/Kreise*), in which urban countries usually represent large cities with more than 100,000 inhabitants or medium-sized cities between 20,000 and 100,000 inhabitants. We have calculated the proportion of the total population in urban and rural counties that was covered by the counties with services that distributed drug paraphernalia in 2021. The study questionnaire was distributed online and by postal service. The online questionnaire was developed using the software VOXCO version 6.0.0.51. All services which participated in the first cross-sectional study were contacted both via postal service containing a hard copy and via e-mail containing a link to the online survey. All remaining services in the database were contacted via e-mail only, for resource reasons. The link to the online survey was also available on the study homepage [23] and was included in different newsletters targeting drug services in Germany (snowball sampling). Up to two reminders were sent via e-mail. When clarification of filled-in questionnaires was needed, services were contacted individually, if contact details were provided.

Inclusion criteria for the services were the distribution of any kind of drug paraphernalia in 2021 as well as consent with the terms and conditions of the study.

We conducted descriptive statistical analysis of the cross-sectional data from 2021. For the analysis of the types and quantities of distributed drug paraphernalia, we included only services which distributed at least n/s or LDS items. We calculated the WHO indicator by dividing the total numbers of distributed n/s by the total number of PWID supplied by the services (including only observations with n/s and PWID > 0). For the calculation of the WHO indicator for syringes, we added the number of distributed LDS items to the number of distributed “conventional” syringes.

We compared the WHO indicator for services with and without a drug consumption room and for services located in urban and rural counties using the Wilcoxon test. We set the significance threshold for statistical hypothesis tests to 0.05. In order to compare the quantity of distributed drug paraphernalia, the WHO indicator and the budget between 2018 and 2021, services participating in both cross-sectional studies were considered a cohort. To compare the number of distributed drug paraphernalia among services in this cohort, we calculated the median of all changes between the two cross-sectional studies at the individual service level.

We analysed the data with MS Excel, R version 4.1.3 and RStudio version 2022.07.2 + 576. We created the map with R version 4.1.3, the package tmap [24] and the shapefile for Germany of GADM version 4.1 [25].

## Results

### Cross-sectional study 2021

#### Response rate

Of 1760 distributed questionnaires plus snowball sampling, 589 were returned (estimated response rate 33%). Services with invalid responses (*n* = 55) or which did not distribute drug paraphernalia (*n* = 330) were excluded. For further analysis, we included 204 drug services from 15 out of 16 federal states in Germany, which confirmed drug paraphernalia distribution. Of all included drug services, *n* = 180 specified the type and quantity of distributed drug paraphernalia (see flow chart, Additional file 1). 108 services had also participated in 2018 and are further on referred to as the cohort.

#### Geographical distribution of services

The drug services with available address information (*n* = 196), were located in 15/16 federal states and 113/400 counties in Germany. The number of distributing services differed between *n* = 76 and *n* = 1 between states.

57% (111/196) of services were located in urban counties, accounting for:

- 51% (54/106) of urban counties, and
- 27% of the urban population.

43% (85/196) of the services were located in rural counties, accounting for:

- 20% (59/294) of rural counties, and
- 21% of the rural population.

**Fig. 1:**
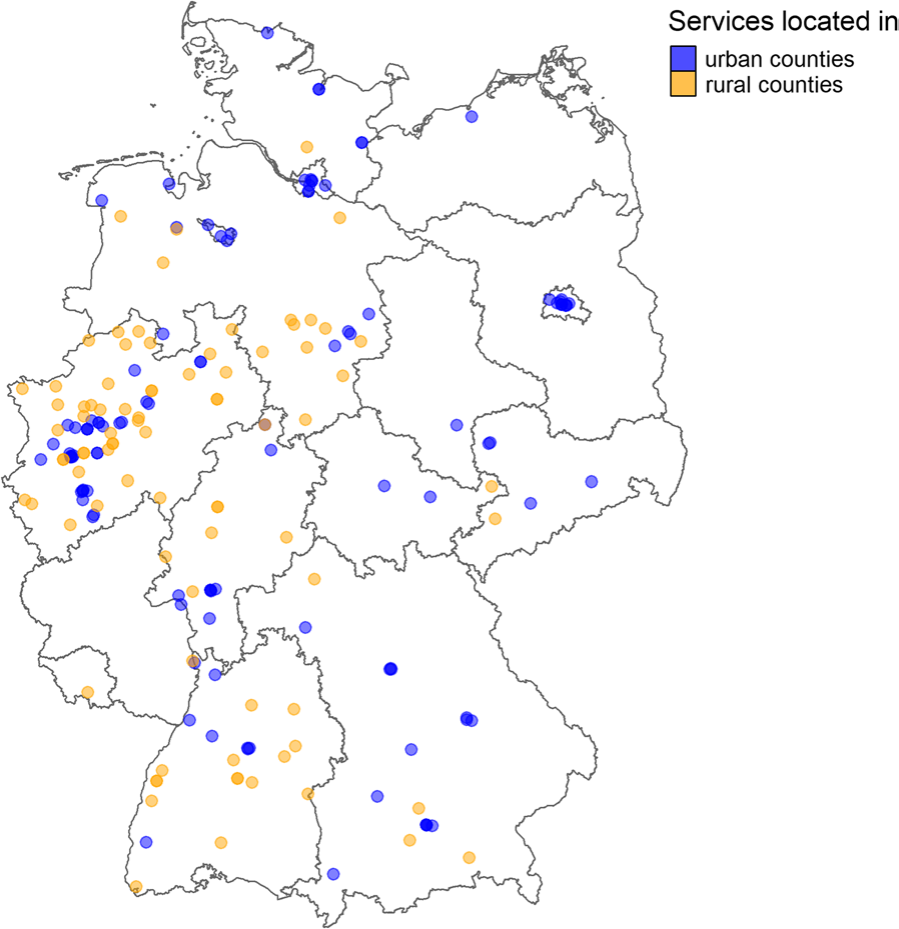
Participating drug services in Germany with available geographical information, 2021 (*n* = 196). Map produced in January 2023. Administrative boundaries: GADM version 4.1 (https://gadm.org/index.html, last accessed 20 January 2023)

The geographical distribution shows that more services were located in the West compared to the East of Germany (see Fig. 1).

#### Distribution sites and persons reached

Of *n* = 203 services with a valid answer on one or multiple distribution sites, 50% stated that they distributed drug paraphernalia via low-threshold services followed by drug counselling centres (41%), outreach (38%), vending machines (33%), drug consumption rooms (12%) and housing projects (5%) (multiple answers possible). 8% of the services indicated drug paraphernalia distribution via additional sites, such as party settings. Distribution of n/s via syringe vending machines was reported by services in 9 states.

Of all drug services that reported more than one distribution site (*n* = 110), half reported that they distributed the majority of their paraphernalia via low-threshold services, followed by drug counselling centres (15%), vending machines (13%) and outreach (11%).

Drug services which (also) had a drug consumption room (*n* = 18) supplied on median almost nine times as many PWID in 2021 than services without a drug consumption room (*n* = 125) (600 vs 70 PWID).

#### Distribution mode and costs of distributed n/s

Of *n* = 188 services which answered the question on the distribution mode of drug paraphernalia, 77% stated to distribute drug paraphernalia without limiting the number of pieces per client, while 37% of services offered n/s in exchange for used materials and 18% of services stated to distribute a restricted number of paraphernalia per client; 32% distributed via vending machines (multiple answers possible).

Of *n* = 94 services offering more than one distribution mode, 21% stated distributing both according to individual need as well as restricting the number of drug paraphernalia per client. This apparent contradiction is discussed below. On federal state level, results were heterogenous: While in 6/15 states accounting for *n* = 16 services, 100% of services stated to (also) distribute according to individual need, in two states this was reported by only 43% and 50% of the services (*n* = 4).

Of *n* = 181 services, 82% distributed n/s for free, while one-quarter of services sold them at cost price and 4% at prices higher than the cost price (distribution via vending machines excluded; multiple answers possible). At the federal state level, the proportion of services, which distributed n/s for free, ranged between 58% (1 state accounting for *n* = 7 services) and 100% (9 states accounting for *n* = 31 services).

#### Types and quantities of distributed drug paraphernalia

N/s were distributed by nearly all of the participating services, followed by vitamin C (155/172, 90%) and pre-cut aluminium foil (134/169, 79%) (Table 1). Overall, drug paraphernalia for injecting consumption were most frequently distributed. A total of *n* = 36 services in 9 federal states stated that they distribute LDS items. Condoms for safer sex were distributed by 92% of the services (159/173).

**Table 1:** Types of drug paraphernalia distributed by participating drug services, Germany, 2021 (*n* = 180)

Drug paraphernalia	Responding services (*N*)	Distributing services (*n* (%))
***Injecting use***		
Syringes (regardless of size; excluding low-dead-space syringes)	174	168 (97%)
Needles (regardless of size)	174	167 (96%)
Low-dead-space items	154	36 (23%)
Spoons	164	127 (77%)
Filters	162	121 (75%)
Vitamin C	172	155 (90%)
Water	161	111 (69%)
Vein ointment	151	53 (35%)
***Inhaling***		
Aluminium foil	169	134 (79%)
Pipes^a^	156	43 (28%)
Pipe screens^b^	156	25 (16%)
Bicarbonate	149	39 (26%)
Mouthpieces	154	17 (11%)
***Nasal use***		
Nasal ointment	143	16 (11%)
Sniff tubes	160	69 (43%)
***Safer sex***		
Condoms	173	159 (92%)
Lubricant gel	156	68 (44%)

^a^Pipes commonly used for crack consumption

^b^A pipe screen is used for crack consumption to hold the rock of crack cocaine in place near the end of the glass stem

On federal state level, results were heterogenous: In 2 states, all 17 drug paraphernalia types were distributed, in 3 federal states no distribution of paraphernalia for nasal consumption was reported and in 5 of the federal states aluminium foil was distributed as the only utensil for inhaling drugs.

Of all the drug paraphernalia for which we assessed the distributed quantities, the median number of distributed n/s was highest (Table 2). The median number of needles distributed (*n* = 5134) was almost 1.5 times higher than the median number of syringes distributed (*n* = 3642). Although the number of responding services for LDS items (*n* = 33) was much lower than for “conventional” syringes and needles, among those services distributing LDS items it was the third most frequently distributed utensil with a median of *n* = 2000. With a median of 100 and 75 pieces, pipes and mouthpieces were the least commonly distributed paraphernalia.

**Table 2:** Quantity of distributed drug paraphernalia by participating drug services, Germany, 2021 (*n* = 180)

Drug paraphernalia	Responding services (*N*)	Median (1st, 3rd quartile)
Syringes (regardless of size; excluding low-dead-space syringes)	160	3642 (653; 15,283)
Needles (regardless of size)	158	5134 (1144; 29,733)
Low-dead-space items	33	2000 (102; 11,489)
Filters	103	1200 (200; 7474)
Spoons	112	800 (120; 3152)
Pipes^a^	41	100 (20; 470)
Pipe screens^b^	22	166 (42; 971)
Mouthpieces	14	75 (39; 173)
Sniff tubes	57	120 (40; 400)

^a^Pipes commonly used for crack consumption

^b^A pipe screen is used for crack consumption to hold the rock of crack cocaine in place near the end of the glass stem

Nearly half (85/176, 48%) of the services estimated the amount of distributed paraphernalia.

#### WHO indicator

On average, 85 syringes and 127 needles were distributed per PWID supplied by the participating services in 2021. On federal state level, 2 states reached the WHO 2030 target for needles and 1 state the 2030 target for syringes. The WHO 2020 target for needles and syringes was reached by 6 and 4 states, respectively. The majority of services (121/190, 64%) estimated the number of supplied PWID, which was used to calculate this WHO indicator.

Drug services in urban counties distributed statistically significant more n/s per PWID compared to services located in rural counties: 152 needles per PWID in urban vs 60 in rural counties (*p* = 0.02) and 101 syringes per PWID in urban vs 44 in rural counties (*p* = 0.03). When stratifying for services without drug consumption rooms, which are mostly located in urban counties, a statistically non-significant difference in the number of distributed n/s between urban and rural counties remained: 138 needles per PWID in urban counties vs 67 in rural counties (*p* = 0.08) and 95 syringes per PWID in urban vs 53 syringes in rural counties (*p* = 0.06).

Services with a drug consumption room distributed statistically significant more needles per PWID compared to services without a drug consumption room: 138 vs 115 needles (*p* = 0.01); no statistically significant difference could be detected for syringes (89 vs 81 syringes, *p* = 0.12).

#### Budget

The responding services (*n* = 105) had a median budget of 2000€ in 2021 for the distribution of drug paraphernalia. Nearly one-third of services assessed their budget as not sufficient (48/157, 31%). The majority of services (103/144, 72%) reported that their budget remained stable, while 18% (26/144) reported an increase and 10% (15/144) a decrease of the available budget for drug paraphernalia since 2018.

#### Cohort 2021

Comparing the quantities of drug paraphernalia distributed by services participating in both cross-sectional studies (*n* = 108) showed a median reduction of 18% for distributed syringes and 12% for needles, respectively (Table 3). For sniff tubes we observed a median reduction of 22%. Among the small number of services which quantified the number of distributed LDS items (*n* = 6) and pipes (*n* = 4) in both 2018 and 2021, the median distribution of LDS items increased markedly (+ 193%) and more than doubled for pipes (+ 214%). For the quantity of distributed filters and spoons no change occurred between 2018 and 2021.

**Table 3:** Comparison of quantity of distributed drug paraphernalia by drug services participating both 2018 and 2021

Drug paraphernalia	Responding services (N)	2018	2021	Median change (1st; 3rd Quartile)	Median change^b^ (%)
		Median (1st; 3rd Quartile)	Median (1st; 3rd Quartile)		
Syringes (regardless of size; excluding low-dead-space syringes)	82	6917 (1341; 30,752)	4794 (975; 20,150)	−328 (−7175; 1000)	−18* (*p* = 0.04)
Needles (regardless of size)	79	10,648 (2506; 46,647)	7600 (2000; 33,600)	−761 (−10,554; 1000)	−12 (*p* = 0.11)
Low-dead-space items	6	9158 (1900; 31,604)	9643 (750; 48,513)	+ 3885 (438; 40,434)	+ 193 (*p* = 0.22)
Filters	40	4422 (1075; 24,262)	4250 (1016; 13,912)	0 (−2850; 964)	0 (*p* = 0.64)
Spoons	40	1856 (463; 9174)	2000 (488; 7200)	0 (−1614; 863)	0 (*p* = 0.88)
Pipes^a^	4	375 (325; 400)	900 (718; 2730)	+ 600 (355; 2443)	+ 214 (*p* = 0.13)
Sniff tubes	12	596 (200; 3375)	400 (260; 1443)	−125 (−695; 185)	−22 (*p* = 0.39)

^a^Pipes commonly used for crack consumption

^b^*p*-values based on binomial test for relative changes, * = significant *p*-value (< 0.05)

Responses from the services participating in both 2018 and 2021 showed that the median number of supplied PWID per service decreased over time (200 in 2018 vs 150 in 2021).

In services included in both surveys, we found a decrease in the median number of distributed syringes and needles per supplied PWID: 100 needles and 73 syringes in 2021 compared to 159 needles and 102 syringes per PWID in 2018.

The median budget for services within the cohort (*n* = 43) increased from 3000€ in 2018 to 4500€ in 2021. On level of the individual services in the cohort, the budget increased for half of the services (22/43, 51%), while it decreased for 35% (15/43) and remained stable for 14% (6/43). The proportion of services assessing their budget as not sufficient among this cohort increased from 33% (20/61) in 2018 to 44% (27/61) in 2021.

## Discussion

### Drug paraphernalia distribution in 2021 and changes since 2018

With this second national survey of drug services we reached 204 distributing drug services in 15 out of 16 federal states and were able to assess the current situation of drug paraphernalia distribution in Germany and changes since 2018. In 2021, the vast majority of included services distributed drug paraphernalia for injecting use. While pre-cut aluminium foil was distributed by nearly 80% of the services, other drug paraphernalia for inhaling and paraphernalia for nasal use were distributed less frequently.

We found heterogenous trends in drug paraphernalia distribution comparing the cross-sectional data from 2021 with results from 2018. Our data showed a decrease of distributed n/s and of sniff tubes among cohort-services between 2018 and 2021, while the numbers of distributed filters and spoons remained stable, and the distributed LDS items and pipes increased. Although the median reduction of 18% for syringes was the only significant change over time, the decreasing trend for needles are no less cause for concern. The decreasing number of distributed n/s might reflect a current trend in Germany towards more inhaling of drugs with a simultaneous decrease in injecting use both individually and at the population level [17, 26, 27]. We saw a comparatively high and increasing number of distributed LDS items and services from the majority of federal states reporting distribution of LDS items, pointing towards a positive trend of implementing the use of LDS items as additional harm reduction intervention. This trend might be especially relevant for areas and regions with an increased risk of n/s sharing, e.g. due to a low availability or accessibility of drug services. The increased distribution of pipes between 2018 and 2021 should be interpreted in the context of an increase in crack cocaine use within the open drug scene, which was recently reported both at the European level and in a number of regions in Germany [28–31].

Our observed changes might also partly be explained by various effects of the COVID-19 pandemic, especially the decrease in n/s distribution. A study conducted in May 2020 on the impacts of the COVID-19 pandemic on users of low-threshold drug services in Germany revealed an overall trend towards reduced opening hours of the services and interim closures during the lockdown in the first year of the pandemic. Other adaptation measures of drug services during these first months of the pandemic included the reduction of consumption places and communal spaces within the services and an increase of outreach activities. Services reported increased financial constraints of PWID and limited possibilities to spend time in the services [32, 33]. A decrease of harm reduction services and a disruption of street drug markets have also been described for the early phases of the pandemic by other European countries [34, 35]. Although these observations from 2020 might not be completely transferable to 2021 as our studied period, certain effects of the first phase of the COVID-19 pandemic on the functioning of drug services surely remained [33] and were also informally reported to the study team by several participating drug services: staffing shortages, remaining efforts to reduce close contacts, and increased prices for and temporarily limited availability of disinfectant as well as n/s.

Rising costs for n/s and increased hygiene efforts (e.g. hand disinfection) during the COVID-19 pandemic in 2021 might have contributed to our results on the budget development with an increasing number of services assessing their budget as not sufficient, despite an overall increase of budget among the services in the cohort.

### WHO indicator

Based on our results on national level, Germany is still not reaching the WHO 2030 target with an even smaller number of distributed n/s per PWID than in 2018.

Our estimate of the WHO indicator for Germany allows comparison with data from other European countries. In 14 countries with available data from 2019 or the latest available data, there was a wide range of 1–616 syringes distributed per PWID [20]. Of those, 9 countries distributed more syringes per PWID than Germany in 2021. While, according to our data, Germany has still not reached the WHO target of 200 syringes per PWID by 2020, this target was reached by Luxembourg, Norway and Finland.

### Geographical differences

We observed geographical differences regarding the distribution of drug services. The concentration of drug services was higher in urban than in rural counties. Besides the presence of drug services, also the quantitative supply differed between urban and rural counties with a higher WHO indicator in urban counties. The discrepancy for the WHO indicator might be largely explained due to almost all included drug consumption rooms (20/24) being located in urban countries.

Recent evidence on the geographical distribution of PWID in Germany is lacking. However, the estimated numbers of individuals addicted to opioids based on the registered numbers of individuals undergoing substitution treatment show higher rates of individuals addicted to opioids in states with large cities compared to more rural states [36]. It remains problematic that access to drug services for PWID depends on their geographical location in Germany. This imposes the risk of underserving PWID in rural counties.

### Modes and sites of drug paraphernalia distribution

The German AIDS Service Organization recommends the distribution of drug paraphernalia according to individual need, i.e. not to restrict the number of supplied needles and syringes and not to distribute needles and syringes in 1:1 exchange, in order to reduce the risk of their re-usage and sharing [18]. Thus, it is positive that the majority of services stated that they distribute drug paraphernalia (also) according to individual need.

However, nearly one quarter of services offering more than one distribution mode, reported to distribute both according to individual need but to also restrict the number of drug paraphernalia per client. Different distribution modes within the same service can be related to different modes being implemented for different types of drug paraphernalia and/or changes in the distribution mode over the year. These mixed results show that distribution according to individual need, also for n/s, has not been fully implemented yet in Germany. Moreover, nearly 20% of services reported that they do not distribute n/s for free. Insufficient budget might be a major reason for drug services to restrict the number of distributed drug paraphernalia and to not distribute n/s for free.

Heterogenous results between states regarding the distribution mode (according to individual need vs exchange programmes) might also reflect differences in regional policies.

Besides the distribution of drug paraphernalia, harm reduction services should ideally be embedded in comprehensive programmes including other components such as opioid substitution treatment to fulfil their full harm reduction potential [1, 8].

Our observation that drug consumption rooms were able to supply much larger numbers of PWID with a statistically significant higher number of needles distributed compared to services without drug consumption rooms, again underlines the importance of drug consumption rooms as harm reduction institutions [37–39]. However, drug consumption rooms are available in only half of the federal states of Germany yet.

### Limitations

Due to the limited response rate our results should be interpreted with caution. The proportion of services among the contacted services which are not distributing drug paraphernalia and which might be less likely to respond is unknown. Due to the relatively small sample size in some states, achieving reliable results on subnational level and conducting comparisons between states is challenging. However, as services from all major organizations of low-threshold drug services in Germany participated both in 2018 and 2021 and the urban counties with participating services accounted for more than one quarter of the overall urban population, we nevertheless consider our results an important and valid assessment of the overall situation of drug paraphernalia supply by drug services in Germany.

Due to slight differences in the applied questionnaires of 2018 and 2021, the calculation of the WHO indicator for syringes for 2018 was based on “conventional” syringes only, without LDS items; while for 2021 we used a combined variable including syringes and LDS items to calculate the WHO indicator for syringes. Therefore, we might overestimate the WHO indicator for 2021, i.e. our observed reduction of the indicator between 2018 and 2021 is a conservative estimate.

There is no data on the current number of PWID in Germany. Validity of the number of PWID supplied by the participating services that we used to calculate the WHO indicator is impacted by the high proportion of services which only estimated the number of PWID, leading to possible over- or underestimations which we cannot control for.

When interpreting the results of the cohort, a possible selection bias should be considered, as services being willing and able to participate in both cross-sectional studies might systematically differ from services only participating in one of the cross-sectional surveys (e.g. having more financial and human resources).

As we focused our study on low-threshold drug services in Germany, we were not able to take other sources for drug paraphernalia supply into account, such as the internet or pharmacies, which might also play a role for drug paraphernalia supply.

## Conclusions

Our study provides cross-sectional data on the geographical coverage of drug services, the quantity and variety of distributed drug paraphernalia, the WHO indicator and the budget of drug services. This is important for an assessment of the current situation of drug paraphernalia supply in Germany and future policy planning. A comparison to the results of the initial cross-sectional study in 2018 on the quantity of distributed drug paraphernalia and the WHO indicator are key to assess the progress of Germany in reaching national and international harm reduction targets. In comparison to other European countries with available data, Germany lies in the lower middle range regarding the WHO target for 2020.

Based on our data, Germany has still not reached the WHO target; however, strong regional and local differences exist. To achieve the WHO target until 2030, supporting the expansion and investing in drug services seems important. Alternative models of accessing clean drug consumption materials need to be assessed, in particular for rural regions. Our data underlines again the importance of drug consumption rooms, both in terms of the number of reached PWID and the number of supplied needles and syringes per PWID. Drug consumption rooms play an important role for harm reduction strategies and should be available in all regions with a need.

Barriers for free distribution of n/s, e.g. on financial or policy level, should be further explored in order to overcome them. As nearly one-third of drug services assessed their budget as insufficient, mechanisms to overcome this financing gap and ensuring a supply, which meets the demand of PWID are needed.

Geographical differences in drug paraphernalia supply should be further investigated and evened out, where necessary, as access to adequate and sufficient drug paraphernalia should not depend on the geographical location of the person who injects drugs. In rural counties with smaller number of PWID innovative approaches for drug paraphernalia supply, such as postal dispatch of package sizes sufficient for one or more weeks, should be considered. In geographical regions and community settings with especially high risk of sharing and reusing n/s, drug services should consider promoting the use of LDS items and to further explore their potential.

A possible change in consumption patterns, especially an increase of inhaling drugs, should be closely monitored. This question could be included in further follow-up studies. Considering potential changes in drug consumption patterns also underlines the importance of additional harm reduction indicators besides the WHO indicator on n/s.

We assessed the overall number of supplied PWID by each service without specifying the frequency of individual drug consumption. Further studies focusing on individual coverage of PWID, defined as the percentage of injecting episodes in relation to the number of sterile n/s received [40], are needed to better assess the coverage with sterile drug paraphernalia according to each individual’s needs.

More alternative drug distribution sites, such as party settings, might become more relevant in the future, especially for certain target groups (e.g. men who have sex with men, chemsex users) [41] and thus should also be explicitly targeted in future studies.

Various impacts of the COVID-19 pandemic, e.g. on consumption patterns, consumption sites, access to drug services, increasing prices or interim supply shortages of n/s might have contributed to our observation of a reduction of supplied PWID, distributed n/s and a decrease of the WHO indicator. Further studies are needed to assess if our results, especially the reduction of distributed n/s, missing the WHO target by far and insufficiency of available budget, were a one-off decline impacted by the COVID-19 pandemic or rather part of a long-term trend.

### Supplementary Information

**Additional file 1**. Flow chart of the cross-sectional study on drug paraphernalia distribution in Germany 2021

## Data Availability

The study questionnaire (in German) and datasets used and analysed in the current study are available from the corresponding author on reasonable request.

## Abbreviations

WHO: World Health Organization
n/s: Needles and syringes
PWID: Person who inject drugs
LDS: Low-dead-space needles; low-lead-space syringes
