# Supplementary material for: International harm reduction indicators are still not reached: results from a repeated cross-sectional study on drug paraphernalia distribution in Germany, 2021
Source: Harm Reduct J. 2023 Sep 19;20:137. doi: 10.1186/s12954-023-00870-2 (PMC10507885; doi:10.1186/s12954-023-00870-2)
Supplement: Supplementary file 1 — Additional file 1. Flow chart of the cross-sectional study on drug paraphernalia distribution in Germany 2021 [file 12954_2023_870_MOESM1_ESM.pdf]

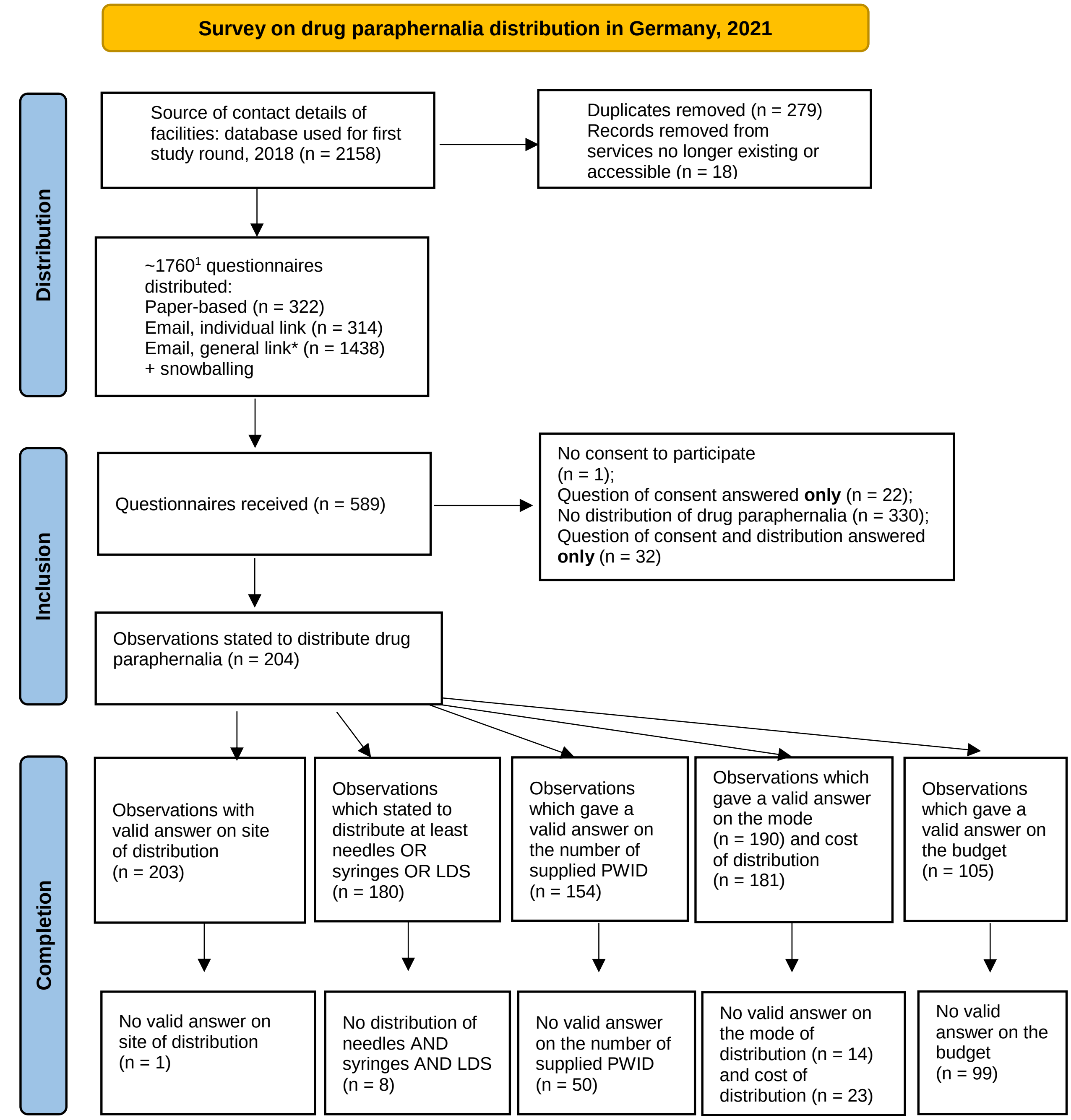

PWID = person who inject drugs; LDS = low-dead-space needles/syringes

[1] Services which had the same email and/or postal address only received the questionnaire once. Thus, the number of distributed questionnaires is less than the number of entries in the initial database minus the duplicates and non-reachable services.

* Emails which were undeliverable were newly researched and resent. However, some emails remained undeliverable. Thus, not all of the emails sent reached the respective services.
